# Ecstatic or Mystical Experience through Epilepsy

**DOI:** 10.1162/jocn_a_02031

**Published:** 2023-09-01

**Authors:** Fabienne Picard

**Affiliations:** Hôpitaux Universitaires de Genève

## Abstract

Ecstatic epilepsy is a rare form of focal epilepsy, so named because the seizures' first symptoms consist of an ecstatic/mystical experience, including feelings of increased self-awareness, mental clarity, and “unity with everything that exists,” accompanied by a sense of bliss and physical well-being. In this perspective article, we first describe the phenomenology of ecstatic seizures, address their historical context, and describe the primary brain structure involved in the genesis of these peculiar epileptic seizures, the anterior insula. In the second part of the article, we move onto the possible neurocognitive underpinnings of ecstatic seizures. We first remind the reader of the insula's role in interoceptive processing and consciously experienced feelings, contextualized by the theory of predictive coding. This leads us to hypothesize that temporary disruptions to activity in the anterior insula could interrupt the generation of interoceptive prediction errors, and cause one to experience the absence of uncertainty, and thereby, a sense of bliss. The absence of interoceptive prediction errors would in fact mimic perfect prediction of the body's physiological state. This sudden clarity of bodily perception could explain the ecstatic quality of the experience, as the interoceptive system forms the basis for unified conscious experience. Our alternative hypothesis is that the anterior insula plays an overarching role in the processing of surprise and that the dysfunction caused by the epileptic discharge could interrupt any surprise exceeding expectations, resulting in a sense of complete control and oneness with the environment.

## INTRODUCTION

An “ecstatic” experience corresponds to an altered state of consciousness, characterized by a sense of mental clarity and the feeling of an expansion of the self-consciousness, which is associated with physical well-being. The experiencer is suddenly plunged into a different state of consciousness regarding the self and the external world. The frontiers between oneself and the environment disappear as if the self-consciousness was enlarged to include everything. The experiencer may report feelings of revelation, clarity, or understanding of the meaning of life. The experience is similar to what has been called a “mystical experience,” a state that has long fascinated philosophers, psychologists, theologists, and neuroscientists alike. The American psychologist William James defined a mystical experience with the following criteria: (i) *ineffability* in the way the experience “defies expression”; (ii) *noetic quality* (state of knowledge, or insight into depths of truth, unaffected by the discursive intellect; illumination, revelation, full of significance, and importance); (iii) *transiency*: a mystical state cannot be sustained for long; and (iv) *passivity*: the mystical state feels as if the will was in abeyance (James, 1902). The English philosopher Stace argued that mystical experiences from a variety of sources have a “common core” of phenomenological features independent from the interpretation of those experiences (Stace, 1960). He stated that a crucial feature defining the mystical experience is a sense of unity, or the “experience of becoming one with all that exists.” Similarly, the English philosopher and writer Huxley described the mystical experience as the “immemorial and universal” substrate underlying all spiritual paths and reflected in every religious tradition (Huxley, 1947). This experience of oneness between participant and object is described in Buddhism as “nondual” awareness (Josipovic, 2010, 2016).

Interestingly, an ecstatic (or mystical) experience may occur as an epileptic phenomenon. In patients suffering from the rare form of “ecstatic epilepsy,” the first part of seizures is dominated by a “feeling of a conscious self-presence, characterized by a heightened self-awareness, and a feeling of union with the world, the ‘All,’” with a temporary suspension of the barrier between the participant and surroundings (Gschwind & Picard, 2016). In the present perspective article, the phenomenology of the ecstatic epileptic experience will be detailed. A small cohort of patients with ecstatic epilepsy provided testimonials allowing us to highlight the homogeneous phenomenology of the first part of the seizures and dissect the foundations of the blissful state. The *primum movens* of the ecstatic state appeared to be a feeling of absence of doubts (an absence of uncertainty) instead of a primary intense positive emotion. After discussing the place of mystical experience in historical, religious context, and culture, we will provide the arguments demonstrating the major involvement of the brain structure called the insula in this phenomenon. Following that, we will share the current knowledge on the role of the insula in the perception of the body's physiological state (or interoception) and in the construction of emotions, feelings, and the sense of self. Finally, we will propose a neurocognitive model for the ecstatic phenomenon, based on the function of the anterior insula in interoceptive predictive coding processing. An alternative, bold hypothesis will be presented, based on a newly emerging function of the anterior insula in the processing of global surprise, which would account for the feelings of mental clarity and certainty reported during the ecstatic experience.

## THE ECSTATIC EPILEPTIC AURA: DESCRIPTION

Epileptic seizures are defined as “a transient occurrence of signs and/or symptoms due to abnormal, excessive or synchronous neuronal activity in the brain” (Fisher et al., 2017). In focal seizures, the abnormal electrical discharge only affects one part of one cerebral hemisphere at the beginning and can then extend through the brain, whereas in generalized seizures, it affects the whole cortex of both cerebral hemispheres from the start. A focal seizure is a dynamic phenomenon with an evolution of symptoms depending on the temporo-spatial evolution of the discharge within the brain over seconds or minutes. The first symptoms of a focal seizure may only be subjective, not visible to onlookers, constituting the epileptic *aura*. There can be an infinite variety of epileptic auras, as in each patient the seizure depends on the specific group of neurons that are involved by the abnormal discharge: For instance, there can be a feeling of ascending fear from the stomach, a feeling of *déjà vu*, auditory hallucinations, olfactory hallucinations, or “forced thinking” (recurrent intrusive thoughts; Pack, 2019). The epileptic aura always lasts less than 2 or 3 min, usually only between a few seconds and 20–30 sec, and is stereotyped in a given patient. Thereafter, the seizure may or may not evolve into a loss of consciousness and further into a secondary bilateral tonic–clonic seizure. Ecstatic epileptic auras have occasionally been reported in patients suffering from focal epilepsy, most often as single cases (for a review, see Picard & Craig, 2009). In 2016, we reported a total of 52 cases collected in medical literature (Gschwind & Picard, 2016). Thereafter, we observed that the frequency of this type of focal epilepsy with ecstatic symptoms is underestimated, as patients are often reluctant to speak about these symptoms. They do not find the words to express what they experience and fear that they will be mistaken for patients with psychiatric disorders. We published the cases of 10 patients (Bartolomei et al., 2019; Picard, 2013; Picard & Craig, 2009), but have identified about 30 patients in total.

The original understanding of these ecstatic seizures was that the patients experienced a feeling of bliss, that is, highly positive emotions (Picard & Craig, 2009); however, through detailed interviews with patients, we proposed that the *primum movens* was not an intense positive emotion, but a feeling of certainty or clarity (as if everything seemed self-evident, completely coherent), which then gave rise to the serenity and/or bliss (Bartolomei et al., 2019; Picard, 2013). All the reported patients had a feeling of physical well-being, described as a feeling of warmth ascending from the feet to the head or a feeling of the body being covered in velvet. Moreover, they had a feeling of expanded self-consciousness and a feeling of revelation (or clarity/evidence/understanding/epiphany) at the time of the seizure. Some patients spontaneously described a feeling of unity with the external environment, whereas others described a feeling of extension of their self, with a loss of boundaries between them and the wider environment, as if there was no separation or individual identity. This is reminiscent of the distinction made by Stace between an extravertive or introvertive mystical experience depending on the qualitative nature of the sense of unity (Stace, 1960). The patients who were still aware of the environment described that all external perceptions were experienced more vividly, with an acute perception of details, such as all outlines and color variety in their visual environment, likely related to a complete experience of the present moment without any thoughts about the past or the future, and to a high level of attention (Picard & Kurth, 2014). All patients described an impression of time dilation with a feeling of an “eternal now” during the ecstatic epileptic phenomenon. Some patients reported that ecstatic experience can be life-changing (e.g., Case 1, not fearing death anymore; Picard & Craig, 2009).

## HISTORICAL CASES AND COMPARISON WITH RELIGIOUS AND OTHER EXPERIENCES

The first description of the ecstatic state in epilepsy dates from Dostoevsky, who openly described his epilepsy in his correspondence to friends and gave the exact timetable of all his epileptic seizures and the description of their immediate consequences on his health in his notebooks (Catteau, 1978). He used the description of his own ecstatic seizures (or what we would now classify as the beginning of his seizures) for characters in his novels, such as his character Prince Mychkine in the novel *The Idiot*, who describes an “ultimate state of harmonious beauty, one that procures an incredible hitherto unsuspected feeling of bliss, appeasement, and understanding of the Supreme Principal of life” (Dostojevskij, 1869). His short novel *The Dream of a Ridiculous Man*, which described the instantaneous transformation of a man, could have been inspired by the experience of his ecstatic auras. The main character of this novel, after a revelation of the “Truth” during a dream, develops a high level of compassion and love for other human beings, and goes on to preach about heaven on earth.

Some of the patients we interviewed described that their experience of ecstatic auras seemed mystical or even “religious.” One of them, an atheist American physicist, shared that he became a Christian after having experienced ecstatic seizures (personal communication).

Descriptions of ecstatic/mystical experiences have been reported by several leading religious figures such as Saint Teresa of Avila (Vercelletto, 1997), Saint Paul (Vercelletto, 1994, 1997), or the Hindu master Ramana Maharshi, all of whom were suspected to have had ecstatic epilepsy. Anecdotally and interestingly, in the 2022 film Avatar 2 by James Cameron, a character called Kiri suffers from an epileptic seizure that induces a “religious ecstasy.” According to the dialogue, an impairment of her “frontal lobe” was suggested. The screenwriters possibly decided that the “frontal” lobe is a more understandable term for the general public than “the insular lobe.”

In Hindu and Buddhist traditions, experiencing a state of unity with all things (i.e., the sense of self is absorbed into something larger) constitutes one of the steps on the path to realization and is most frequently reached through meditation practices focusing on nondual experiences. Some meditation practitioners talk about a state of direct perception or ultimate reality (which is devoid of all our “filters” related to past experiences). In addition, some individuals may have naturally occurring ecstatic/mystical experiences, of non-epileptic origin and outside of all meditation or religious context, often as a single episode in their life (Van der Tempel & Moodley, 2020; Bologne, 2015), sometimes called an “awakening experience” (Taylor, 2012). We can postulate that Albert Einstein agreed on the existence and possible experience of a state of nonduality (everything in the Universe being an inseparable part of it), as he wrote “A human being is a part of the whole, called by us ‘Universe,’ a part limited in time and space. He experiences himself, his thoughts and feelings as something separated from the rest—a kind of optical delusion of his consciousness” (letter to Robert Marcus, 1950). Among other famous people, the French essayist Romain Rolland, laureate of the 1915's Nobel Prize for Literature, described the mystical experience as an oceanic feeling at the source of all religious energy, whereas Sigmund Freud reduced it to the kind of consciousness of an infant of whom the ego has not yet detached from itself the external world. He suggested that this primary ego feeling may survive alongside the narrower and more sharply outlined ego feeling of maturity (*Civilization and Its Discontents*, 1930, translated by James Strachey; Freud, 2015), but he did not give this state a meaning of pure and “original” consciousness.

Mystical experiences induced by psychedelics have been an active area of research these last years, because of their recent medical acceptance as short-term guided treatment, for instance in severe depression (Tullis, 2021; Carhart-Harris et al., 2016). They allow prospective controlled studies of experiences of unity and “recognition of the oneness of all” (Barrett & Griffiths, 2018). Psychedelics that most often induce a complete mystical experience are psilocybin mushrooms, ayahuasca, and peyote (mescaline), under which people describe an expansion of one's own self into the self of others. The 30-item Mystical Experience Questionnaire has been validated in studies of psychedelics as an adequate retrospective measure of mystical experience (MacLean, Leoutsakos, Johnson, & Griffiths, 2012), and was also used in our patients with ecstatic epilepsy (Nencha, Spinelli, Vulliemoz, Seeck, & Picard, 2022). Interestingly, spiritual significance of these experiences under psychedelics was sustained, with positive attitudes about life and self, for more than 1 year after the sessions (Barrett & Griffiths, 2018). However, we consider mystical experiences induced by epileptic seizures and those induced by psychedelics to be different in the sense that mystical experiences induced by psychedelics are not “pure.” A psychedelic-induced mystical experience usually only occurs at high doses, which regularly also induce visual alterations, whether elementary or complex, typically described as hallucinatory phenomena or illusory perceptions (Hirschfeld & Schmidt, 2021; Carbonaro, Johnson, Hurwitz, & Griffiths, 2018). In those cases, we anticipate that the experience will have additional neural correlates than those correlating to the mystical experience alone.

## THE DEMONSTRATION OF THE INVOLVEMENT OF THE INSULA

In prior reports of patients with ecstatic seizures, a dysfunction of the temporal lobe was usually suspected, essentially because some patients had EEG abnormalities (temporal lobe slow focus, spikes, or electrical seizure; Stefan et al., 2004; Asheim Hansen & Brodtkorb, 2003; Naito & Matsui, 1988; Cirignotta, Todesco, & Lugaresi, 1980; Subirana, 1953; Alajouanine, 1951) or lesions in the temporal lobe, particularly tumors, visible on brain computed tomography (Morgan, 1990; Boudouresques, Gosset, & Sayag, 1972; Mulder & Daly, 1952). However, the symptomatogenic zone, responsible for the first clinical symptoms of a seizure, may be different from the epileptogenic zone from where the epileptic discharge starts and spreads, when the epileptogenic zone is clinically “silent,” that is, without any clinical correlate, as it may be for some epileptic electrical discharges starting in the temporal pole.

**Figure 1.**
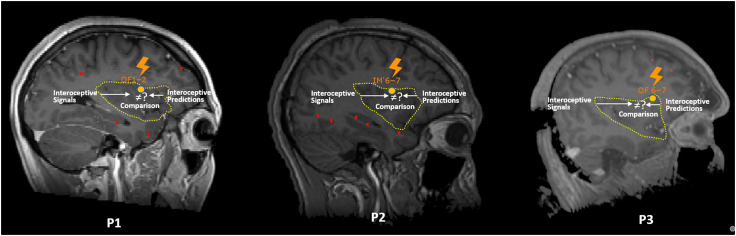
Insular localization (orange circle) of the electrode, where stimulation reproduced the ecstatic aura, in three patients with known ecstatic epilepsy (contacts OF1-2 in Patient 1 (P1); contacts IM'6-7 in Patient 2 (P2); contacts OF6-7 in Patient 3 (P3)). The limits of the insula are indicated with a yellow dotted line. Red dots correspond to other electrodes. In all three patients, stimulations triggering ecstatic auras were at locations within the frontal opercular-anterior insular region (dorsal anterior insula). Interoceptive signals arrive in the posterior insula. The dorsal anterior insula produces interoceptive predictions and generates interoceptive prediction errors by comparing predictions to the real incoming signals. During stimulation (as well as during a spontaneous seizure), we postulate that an interruption of the production of interoceptive prediction errors in the region of stimulation is at the origin of the ecstatic phenomenon. Adapted from a Figure published in *Brain Stimulation* (12(5) (2019), F. Bartolomei, S. Lagarde, D. Scavarda, R. Carron, C. G. Bénar, and F. Picard, The role of the dorsal anterior insula in ecstatic sensation revealed by direct electrical brain stimulation). Reproduced with permission from Elsevier.

Given the observed symptoms in the ecstatic phenomenon, in particular physical well-being and feeling of increased self-awareness, we hypothesized the involvement of the insula (Picard & Craig, 2009), because of its known role in interoception, that is, the perception of the physiological state of the body, and in self-consciousness (Craig, 2009; see next section). In two patients suffering from epilepsy with ecstatic seizures, the results of the ictal SPECT performed during a seizure with ecstatic symptoms showed an increased blood flow in the operculo-anterior insular region (Picard, 2013; Picard & Craig, 2009). Later, we found stronger evidence in favor of the involvement of the anterior insula in the ecstatic phenomenon, in a patient with ecstatic epilepsy undergoing presurgical evaluation with intracerebral electrodes (stereo-EEG). During spontaneous recorded seizures, the ecstatic symptoms were concurrent with the propagation of the electrical discharge from the mesiotemporal region to the dorsal (i.e., superior) anterior insula, within 1 sec. Moreover, her ecstatic auras could be reproduced via electrical stimulation of this part of the insular region, namely, the very dorsal part of the anterior insula (frontal opercular-insular junction; Picard, Scavarda, & Bartolomei, 2013). Stimulations of electrodes in the mesiotemporal region or in any other sampled region did not induce the ecstatic aura. This specific result has been repeated in several patients. In fact, since this first patient's report, ecstatic auras were (i) reproduced by the electrical stimulation of the dorsal anterior insular region in at least six patients with ecstatic epilepsy (personal communication, five at the University Hospital of Marseille and one in London), of which three were reported (Bartolomei et al., 2019; Figure 1), as well as (ii) induced by the electrical stimulation of the dorsal anterior insular region in one patient (at the University Hospitals of Geneva) with temporal lobe epilepsy who had never had ecstatic phenomena previously (Nencha et al., 2022). In all patients, the stimulation of the other electrodes, particularly in the temporal lobe, did not induce an ecstatic aura. In other patients, the stimulation of the (left) amygdala would sometimes induce some pleasant sensations, but never an ecstatic state (Lanteaume et al., 2007). It must be specified that the induction of an ecstatic aura through the stimulation of the dorsal part of the anterior insula is not the rule, as there are many patients in whom stimulations of this region did not give rise to ecstatic experience.

It must be noted that patients with ecstatic seizures could have right or left brain lesions or EEG abnormalities, without any clear argument for a particular lateralization of the ecstatic phenomenon. In the 2016 study, we noted 16 left cases, 21 right cases, and 12 cases with undefined lateralization (Gschwind & Picard, 2016; see Table 1). There is probably an involvement of both insulae during the ecstatic experience as there are rapid connections between both insulae (Lacuey et al., 2016) and thus a likely rapid propagation of an insular discharge to the contralateral insula in most patients.

**Table 1.** Lateralization of the Epileptogenic Region in Patients with Ecstatic Auras Already Reported in Literature

	*Left Lateralization*	*Right Lateralization*
Gschwind and Picard (2016)	16	21
Bartolomei et al. (2019)	1	2
Nencha et al. (2022)	1	
Total number of cases	18	23

The cases of Bartolomei et al. (2019) also had ecstatic auras reproduced by the electrical stimulation of the anterior insula. In the last case (Nencha et al., 2022), ecstatic auras were induced by the electrical stimulation although the patient had never had ecstatic symptoms before.

Recently, there was a report of a time dilation illusion following the electrical stimulation of the right mid-dorsal insular region, during the functional mapping of a patient with drug-resistant epilepsy in the context of a presurgical evaluation (Sheikh, Koubeissi, Spencer, & Alkawadri, 2022). The patient described a feeling “like an eternity” comparable to a feeling experienced as a teenager “while ‘tripping’ with lysergic acid diethylamide (LSD) and mushrooms,” which could correspond, in our opinion, to an ecstatic/mystical experience.

Interestingly, the involvement of the insula could also be shown during a mystical experience related to an ayahuasca session, with increased blood perfusion in the bilateral anterior insula in a SPECT study using ethylcysteinate dimer and statistical parametric mapping analysis between ayahuasca and placebo scans (Riba et al., 2006).

## NEUROCOGNITIVE UNDERPINNINGS

We will consider two possible hypotheses for the occurrence of an ecstatic phenomenon in anterior insular cortex epilepsy. Our primary hypothesis is that the processing of interoceptive predictive coding in the insula is blocked during the ecstatic seizures. We will also consider an alternative hypothesis based on a potential role of the anterior insula in the processing of surprise. Both hypotheses will be discussed in detail in this section.

Interoception is the sensing of the internal physiological state of the body, or perception of the signals coming from inside of the body (Craig, 2002). The posterior insula harbors the primary cortical representation of interoceptive signals (Seth, Suzuki, & Critchley, 2011; Craig, 2002). Craig postulated that there is a re-representation of the internal signals within the insula, from its posterior part to its anterior part, with the integration of other information from limbic and frontal cortices, up to their ultimate representation as consciously experienced feelings in the anterior part of the insula (Craig, 2009).

This view has recently been extended with insights from the theory of predictive coding, which postulates that the brain is constantly making predictions about the causes behind bodily (interoceptive) and sensory (exteroceptive) signals based on a generative model of the world. Top–down signals (predictions) are produced at different hierarchical levels of the brain, for all its systems (interoceptive, visual, auditory, tactile, cognitive, …), and at different time scales, for example, longer for cognitive functions or regulation of some physiological mechanisms such as hunger (for rodents, see Livneh & Andermann, 2021). When the real bottom–up signal arrives in the brain, it is compared with the prediction, with different weights put on the prediction and the real signal, modulated by precision, which changes depending on each signal and on the context (Picard & Friston, 2014). The mismatch between the prediction and the real incoming signal provides a prediction error. In terms of interoception, it is postulated that the anterior insula generates interoceptive predictions and behaves like a comparator module of interoceptive predictions and real incoming interoceptive signals (Figure 1), giving rise to interoceptive prediction errors. Prediction errors can then change the generative model (through updating predictions), or the person's behavior; for example, the person moves to better match the perceptive signal (Seth, 2013; Seth & Critchley, 2013; Seth et al., 2011). The anterior insula is thus thought to generate higher order interoceptive predictions errors by computing the difference between predictions and the real signals arriving from the dorsal insula (Fermin, Friston, & Yamawaki, 2022; Seth, 2013). Emerging evidence on the role of the insula in generation of interoceptive predictions or computation of predictions comes from studies looking at neurophysiological signatures of interoceptive predictions and prediction errors (Fazeli & Buchel, 2018; Geuter, Boll, Eippert, & Büchel, 2017; Allen et al., 2016; Preuschoff, Quartz, & Bossaerts, 2008). A recent fMRI study manipulated breathing resistance to show breathing-related interoceptive predictions and prediction errors in the anterior insula (Harrison et al., 2021).

There is converging evidence in favor of a model in which the insula integrates not only interoceptive signals, but also exteroceptive signals, thereby generating interoceptive predictions that account for exteroceptive predictions, allowing for better anticipation of interoceptive changes caused by the external world. The insula thus provides a multisensory representation of the world from the perspective of an embodied person (a person with a body). The term Embodied Predictive Interoception coding was proposed for the process whereby the prediction signal from the anterior (agranular) insula is sent to the middle and posterior (granular) insula, to be confronted there with interoceptive afferences (Barrett & Simmons, 2015). Furthermore, Fermin and colleagues proposed a model called Insula Hierarchical Modular Adaptive Interoception Control for the formation of higher order interoceptive representations, with different insula modules forming parallel networks (Fermin et al., 2022). The insula creates generative interoceptive models that explain the incoming interoceptive signals and regulate visceral responses according to the demands imposed by the body and environment, with the ultimate goal of allostatic (control-oriented) regulation of physiological states (Seth & Tsakiris, 2018). With a mouse model, Livneh and Andermann showed that insular cortex integrates visceral (interoceptive) and sensory (exteroceptive) signals to compute a prediction of the future physiological state, with distinct interoceptive concomitant predictions likely merging into a single integrated interoceptive prediction (Livneh & Andermann, 2021). In humans, constant interaction between interoceptive and exteroceptive signals has emerged in studies such as that of Critchley and Garfinkel (Critchley & Garfinkel, 2017) showing that images of fearful faces were judged to be more intense if presented during the systolic phase of participants' heart beats. Clearly, unconscious interoceptive processing matters for the (emotional) perception/interpretation of external signals. In a study of interoceptive integration in the context of listening to an emotionally salient audio narrative, Nguyen and colleagues found evidence that the anterior insula facilitates the integration of interoceptive states with exteroceptive signals to highlight emotional moments (Nguyen, Breakspear, Hu, & Guo, 2016).

As mentioned above, through its role in interoceptive representation, the anterior insula is ideally positioned to form the basis for all consciously experienced feelings (Fermin et al., 2022; Seth, 2013; Craig, 2009; Critchley, Wiens, Rotshtein, Ohman, & Dolan, 2004). The role of the anterior insula in explicit representation of feeling states has even been suggested to underlie a conscious representation of self (Seth et al., 2011; Critchley et al., 2004). The idea that the sense of self would be grounded in representations of bodily signals had been previously proposed by Craig (2002) and in Damasio's somatic-marker hypothesis (Damasio, 2003). This idea is in line with the essence of the old James-Lange theory of emotion in the 1880s, which emphasized the importance of the physiological (bodily) changes for emotion. A specific study of subjective feeling states arising from representations of bodily responses (visceral responses accessible to awareness) demonstrated the involvement of bilateral insulae and of the right frontal opercular cortex (Critchley et al., 2004). Altogether, this implies that our perception of the world, and of our self within it, happens through our living body. Interoceptive representations are recognized as an essential part of every mental event (Barrett & Simmons, 2015). The interoceptive system would thus form the basis for unified conscious experience.

Regarding the ecstatic/mystical state related to ecstatic epilepsy, our first neurocognitive hypothesis is that an interruption of the predictive coding system in the field of interoception is at the origin of the ecstatic/mystical experience (Picard & Friston, 2014; Picard, 2013), as it may mimic perfect prediction of the physiological state of the body. We have postulated that the epileptic discharge that affects the anterior insula in patients with ecstatic epilepsy will impede the insula from having the adequate level of complexity necessary for the generation of interoceptive predictions and/or of interoceptive prediction errors (Bartolomei et al., 2019). The next issue is how an absence of interoceptive prediction errors in ecstatic epileptic seizure can explain not only a sense of perfect physical well-being, but also a feeling of a perfect external world, as if encountering the “ultimate reality.” The fact that we are “embodied human beings” (Thompson & Varela, 2001) and that the internal signals are always “contextualized” by and processed with the external signals when they arrive in the insula explains that the moment-by-moment detection of the external world is constantly associated with bodily responses and changes in the physiological state of the body, as mentioned above. In the ecstatic state, a person would feel the physiological state of the body as if she had predicted it perfectly. Our model proposes that such sudden clarity in the perception of the body could explain the ecstatic quality of the experience, with a sense of certainty and unity, potentially ignoring what is truly happening with the environment, and resulting in the feeling that “everything is alright.”

Our second, alternative hypothesis involves a direct role of dorsal anterior insula in surprise processing. The constant activation of the dorsal anterior insula in a large range of tasks (sensory, emotional, cognitive; Chang, Yarkoni, Khaw, & Sanfey, 2013; Kurth, Zilles, Fox, Laird, & Eickhoff, 2010) and its heavy connectivity to a broad range of cortical and subcortical brain regions (Dionisio et al., 2019; Gogolla, 2017) support an overarching role of the insula in the brain's global functioning, such as an integrative role in surprise detection. Although the role of the insula in risk, that is, uncertainty with known probabilities, has been known for a long time (Preuschoff et al., 2008; Kuhnen & Knutson, 2005), Singer and colleagues proposed a broader role of the insula in uncertainty processing (Singer, Critchley, & Preuschoff, 2009). Processing information about uncertainty could be shared across modalities, reflecting predictions and prediction errors related to uncertainty (e.g., pain, taste). The authors postulated that the computation of levels of uncertainty for signals across different modalities, that is, the computation of sensory uncertainty, is expressed as a feeling state (Singer et al., 2009). For instance, the involvement of the anterior insula in the processing of uncertainty has been reported in a task of strategic thinking (Nagel, Brovelli, Heinemann, & Coricelli, 2018), or in the surprise related to visual perception in a task involving the bistable figure of Necker's cube (Loued-Khenissi, Pfeuffer, Einhäuser, & Preuschoff, 2020). A recent fMRI study, using a whole brain search, modeled the BOLD signal as a function of the degree of uncertainty in an orientation stimuli visual task (Geurts, Cooke, van Bergen, & Jehee, 2022). It showed that the dorsal anterior insula (in association with dorsal cingulate cortex and rostro-lateral prefrontal cortex) tracks fluctuations in uncertainty and mediates the link between sensory uncertainty and the confidence expressed. Moreover, it is noteworthy that human disorders such as anxiety disorders and uncertainty intolerance are associated with increased activity in the dorsal anterior insula (Gorka, Nelson, Phan, & Shankman, 2016; Alvarez et al., 2015).

**Figure 2.**
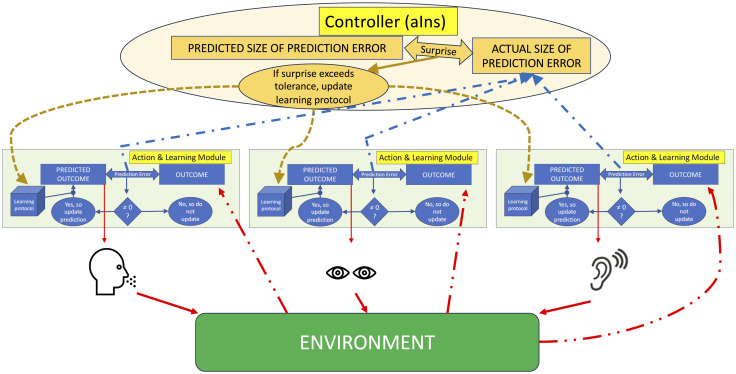
Scheme of our alternative hypothesis: A model proposing the role of the dorsal anterior insula (aIns) as a controller of “unexpected surprise.” The brain consists of many learning modules each updating when there is a mismatch between a prediction and an outcome coming from the environment (or from the body for the interoceptive learning module). Here, three exemplar learning modules are shown (speech, eye gaze, and audition). The anterior insula (aIns) controls the learning modules based on surprise: it compares the acceptable size of a prediction error (“uncertainty prediction”) with its actual size. If the amplitude of the prediction error is higher than its predicted size (“uncertainty prediction error”), there will be a surprise signal, which leads to an update in/a change of the learning protocol. The scheme allows for the possibility that aIns integrates the size of multiple prediction errors into a signal of global surprise. If global surprise surpasses expected surprise, it can inform all the learning modules at once that something fundamental has changed. We hypothesize that the latter is formed by interoception and updated using single integrated interoceptive prediction errors.

As the anterior insula seems to play a role in awareness of uncertainty and surprise, Bossaerts has adapted a theory of model-reference adaptive control (Nguyen & Nguyen, 2018), and suggested that the human brain uses such a model with the anterior insula playing the role of controller (Bossaerts, 2018). The anterior insula would perform surprise computation with which it could regulate learning by other parts of the brain that guide inference (updating of models) and action (active inference) (Picard, Bossaerts, & Bartolomei, 2021; Bossaerts, 2018; see Figure 2). Every fraction of a second, we would experience/interpret the world through a perceptual best guess, with a global uncertainty prediction within the anterior insula, which could correspond to the prediction of the anticipated size of a single integrated interoceptive prediction error. The anterior insula would track surprise magnitude by processing the predictions of global subjective uncertainty moment by moment. A signal of global surprise would derive from an integrated interoceptive prediction whose mismatch with the real incoming interoceptive signal exceeds the expected uncertainty level (with a magnitude of mismatch above the expected level of mismatch). The surprise magnitude is thus the difference between the expected size of prediction errors according to a reference model, that is, the predicted uncertainty, and the real size of prediction errors, that is, the momentarily perceived uncertainty. In short, there is surprise when a prediction error is larger than expected. It is hypothesized that there would be no need for adaptation as long as prediction errors are no larger than anticipated (Bossaerts, 2018). For instance, if heart rate changes caused by external uncertainty are anticipated (e.g., while watching a thriller movie), the changes will not cause a signal of global surprise (with concurrent emotional imbalance). Consequently, there is no need to change the model of the environment. By contrast, a signal of “unexpected surprise” would occur within the insula (the “controller”) in case of a radical change in the global signal of the environment. Then, the anterior insula would indicate to the different brain systems/learning modules (speech, eye gaze, audition, etc.) to adapt their learning model as something fundamental must have changed (Figure 2). According to Loued-Khenissi and Preuschoff, a surprise may indeed generate back-propagating information that allows a learning module to update/switch, to adjust initial beliefs (Loued-Khenissi & Preuschoff, 2020). The authors postulated that at higher levels of the brain hierarchy, when individual brain systems/learning modules cannot be further updated/switched, one could then experience certitude.

During the ecstatic seizure, the new, alternative hypothesis is that, because of the crucial role of insula in global uncertainty processing, no surprise is recorded, halting the updating of all learning modules.

In our opinion, under either neurocognitive hypothesis (interruption of interoceptive prediction errors or no unexpected surprise), the patient would feel in control and, hence, in harmony with the environment, thereby experiencing ecstasy.

To conclude, the study of patients with ecstatic epilepsy has resulted in the first significant breakthrough in understanding the neural correlates of mystical experience. It has identified the anterior insula as a key structure in the generation of the state. The increasing number of cases (not reported in literature) confirm this location via deep intracerebral electrodes recordings in patients, and the reproduction or induction of ecstatic phenomena with the electrical stimulation of the dorsal anterior insula in several patients provides further supportive evidence. Future perspectives are (i) endeavor to demonstrate the dorsal anterior insula's implication in mystical experiences of other origins (meditation practices, psychedelics); (ii) identify possible dispositional traits that make one susceptible to the ecstatic state, as ecstatic/mystical experience only occur in a subgroup of patients with dorsal anterior insular epilepsy; and (iii) test the hypothesis that interindividual differences in the activity of the anterior insula could be the possible neurological foundation of tolerance to uncertainty in the perceptual and cognitive-affective systems. Ultimately, a better understanding of the role of the anterior insula in ecstatic states could lead to new perspectives of (neuromodulatory) treatment in neuropsychiatric disorders, such as severe depression or posttraumatic stress disorder.

## Acknowledgments

We thank Prof. Peter Bossaerts for fruitful discussions and helpful comments, and Nina Sooter for critical reading of the manuscript.

Reprint requests should be sent to Fabienne Picard, Hôpitaux Universitaires de Genève, Departement des Neurosciences Cliniques, Rue Gabrielle-Perret-Gentil 4, Genève 1205, Switzerland, or via e-mail: Fabienne.Picard@hcuge.ch.

## Author Contributions

Fabienne Picard: Conceptualization; Investigation; Writing—Original draft; Writing—Review & editing.

## Diversity in Citation Practices

Retrospective analysis of the citations in every article published in this journal from 2010 to 2021 reveals a persistent pattern of gender imbalance: Although the proportions of authorship teams (categorized by estimated gender identification of first author/last author) publishing in the *Journal of Cognitive Neuroscience* (*JoCN*) during this period were M(an)/M = .407, W(oman)/M = .32, M/W = .115, and W/W = .159, the comparable proportions for the articles that these authorship teams cited were M/M = .549, W/M = .257, M/W = .109, and W/W = .085 (Postle and Fulvio, *JoCN*, 34:1, pp. 1–3). Consequently, *JoCN* encourages all authors to consider gender balance explicitly when selecting which articles to cite and gives them the opportunity to report their article's gender citation balance.
